# Targeting protein homeostasis with nelfinavir/salinomycin dual therapy effectively induces death of mTORC1 hyperactive cells

**DOI:** 10.18632/oncotarget.16232

**Published:** 2017-03-15

**Authors:** Elaine A. Dunlop, Charlotte E. Johnson, Marie Wiltshire, Rachel J. Errington, Andrew R. Tee

**Affiliations:** ^1^ Division of Cancer and Genetics, Cardiff University, Heath Park, Cardiff, CF14 4XN, UK

**Keywords:** mTORC1, TSC, therapy, nelfinavir, cell death

## Abstract

Uncontrolled cell growth in Tuberous Sclerosis Complex occurs due to inappropriate activation of mechanistic (mammalian) target of rapamycin complex 1 (mTORC1). The current therapy, rapamycin, produced promising clinical trial results, but patient tumours regrow if treatment is discontinued, revealing rapamycin has cytostatic properties rather than a cytotoxic effect. Taking advantage of the enhanced levels of endoplasmic reticulum (ER) stress present in *TSC2*-null cells, we investigated drug combinations producing a cytotoxic response. We found a nelfinavir and salinomycin combination specifically killed *TSC2*-deficient, mTORC1 hyperactive cells. Cytotoxicity was rescued by reducing protein synthesis, either through mTORC1 inhibition or cycloheximide treatment. This indicates that the drug combination targets the cells by tipping the protein homeostasis balance of the already metabolically stressed *TSC2*-deficient cells in favour of cell death. Furthermore, this drug combination also inhibited tumour formation in *TSC2*-deficient cell models and caused tumour spheroid death in 3D culture. Importantly, the 3D assay could differentiate the cytostatic agent, rapamycin, from the cytotoxic nelfinavir/salinomycin combination. Sporadic cancer cell lines with hyperactive mTORC1 signalling were also susceptible to this nelfinavir/salinomycin drug combination. This work indicates that the protein homeostasis pathway is an attractive therapeutic target in both Tuberous Sclerosis Complex and mTORC1-driven sporadic cancers.

## INTRODUCTION

The mechanistic (mammalian) target of rapamycin complex 1 (mTORC1) is a central regulator of cell growth, *via* its control of protein synthesis and other anabolic pathways. The tumour suppressors, Tuberous Sclerosis Complex (TSC)-1 and TSC2, lie upstream of mTORC1 and function together with TBC1D7 to negatively regulate the mTORC1 activator, Ras homolog enriched in brain (Rheb). Mutations in *TSC1* or *TSC2* lead to the rare genetic condition, TSC, where patients develop cysts and tumours in multiple organs due to mTORC1 hyperactivity and uncontrolled cell growth. mTORC1 signalling is also inappropriately activated in a number of sporadic cancers (reviewed in 1). For example, over half of breast cancers demonstrate upregulated markers of mTORC1 activation, such as phospho-S6K1 and phospho-ribosomal protein S6 (rpS6) [2, 3], while overexpression of mTORC1 substrates are also strongly associated with prostate cancer [4]. mTORC1 upregulation in such cancers could be due to mutations in a number of upstream oncogenes and tumour suppressors, including those controlling the PI3K-Akt or MAPK signalling pathways which both converge on mTORC1. mTORC1 activation specifically through TSC loss of function is seen in a proportion of bladder cancer [5], hepatocellular carcinoma [6] and pancreatic neuroendocrine tumours [7].

Rapamycin is an allosteric small molecule inhibitor of mTORC1 and is an effective treatment for TSC angiomyolipomas [8, 9]. However, it has been shown that discontinuation of patient treatment leads to tumour regrowth, indicating that rapamycin functions as a cytostatic agent. Instead of inhibiting mTORC1, an alternative strategy for TSC therapy is to exploit the metabolic vulnerabilities of mTORC1 hyperactive cells, which would instigate a cytotoxic response. For example, *TSC*-deficient cells are highly sensitive to glucose withdrawal and undergo glucose deprivation-induced death [10]. Additionally, inhibition of glutaminase sensitises *TSC2-deficient* cells to heat shock protein 90 (HSP90) inhibition through a mechanism of increased oxidative stress [11]. A potential therapeutic avenue is to exploit the fact that mTORC1 hyperactive cells have enhanced basal endoplasmic reticulum (ER) stress, due to the elevated levels of mTORC1-directed protein synthesis placing a burden on the protein folding capacity of the ER. ER stress activates a protective pathway termed the unfolded protein response (UPR), which aims to downregulate protein synthesis and restore protein folding in order to restore cellular homeostasis. However, following excessive ER stress over a prolonged period, apoptosis is initiated [12]. A key player in the ER stress response is C/EBP homologous protein (CHOP, also called growth arrest and DNA damage inducible gene 153 (GADD153)) [13]. Upon acute ER stress, CHOP expression is strongly enhanced through IRE1α- and PERK-mediated pathways. If homeostasis is not restored and the levels of misfolded proteins remain high, CHOP stimulates a transcriptional programme that instigates cell death [12]. Amongst other genes, CHOP directly activates expression of GADD34 [14], a protein phosphatase 1 (PP1) regulator which causes PP1-mediated dephosphorylation of eIF2α [15]. This releases the translational block, thereby enhancing protein synthesis to activate death-associated mechanisms. Further enhancing ER stress through treatment with ER stress inducing drugs has been shown to selectively induce the death of mTORC1 hyperactive cells [16].

Recently, salinomycin has been identified as a potent mediator of breast cancer stem cell death [17]. Follow up studies by other groups indicate that it also induces cell death in bulk cancer cell lines [18–22]. Salinomycin is a potassium ionophore, but the mechanism by which it induces cell death is not yet clear, with unconventional cell death pathways implicated in its mode of action [18]. Several cell signalling pathways are reported to be altered following salinomycin treatment, including enhancement of ER stress [21], inhibition of Wnt signalling [22] and an impact on autophagy [23]. Salinomycin has been reported to inhibit mTORC1 signalling in breast, prostate and lung cancer cell lines [19, 21]. Interestingly, when *CDH1* expression is inhibited in non-small cell lung carcinoma cells, salinomycin induces more cell death than in their wildtype counterparts as mTOR inhibition is alleviated in these cells [21]. This work implies that cells with a higher level of mTORC1 activity are more sensitive to the cytotoxic drug action of salinomycin.

The current study tests the impact of salinomycin treatment on mTORC1 hyperactive cells. We did this in combination with nelfinavir, as TSC2-deficient cells have previously been reported to be selectively targeted by nelfinavir treatment [16, 24]. Nelfinavir inhibits the human immunodeficiency virus (HIV) retroviral protease and is widely used to treat HIV infection. Studies have revealed that nelfinavir can exert multiple cellular effects, such as induction of autophagy and apoptosis in cancer cell lines [25]. Herein, we examined the synergistic potential of nelfinavir and salinomycin to selectively kill *TSC2*-deficient cells and tested the dependence of mTORC1 hyperactivation as a sensitising mechanism of drug cytotoxicity. We also employed 3D cell spheroid models to measure tumour shrinkage post-drug treatment and further examined the concept of cytotoxic *versus* cytostatic drug action, using DRAQ7 as the unified signal readout of cell viability across all the models from 2D to 3D. Our work highlights the therapeutic potential of ER stress inducers that show selective cytotoxicity in mTORC1 hyperactive cells.

## RESULTS

### Combined nelfinavir and salinomycin treatment induces ER stress

**Figure 1:**
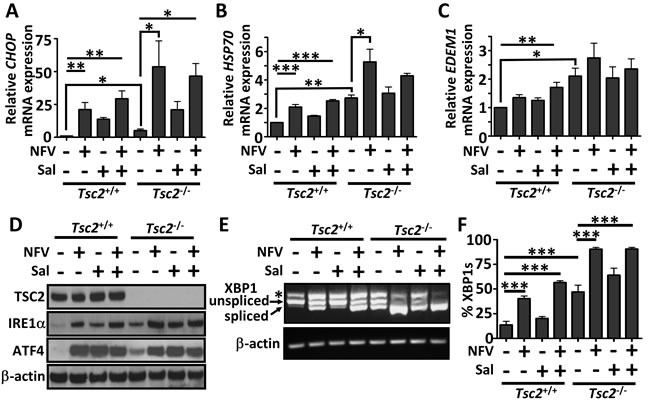
ER stress is elevated by nelfinavir and salinomycin treatment *Tsc2*^+/+^ and *Tsc2*^−/−^ MEFs were treated with either DMSO vehicle, 20 μM nelfinavir (NFV) or 5 μM salinomycin (Sal) as single agents or in combination for 6 h. Gene expression of *CHOP* **A**., *HSP70* **B**. and *EDEM1* **C**. was determined by real time PCR and relative mRNA expression was standardised to β-actin. Cells which had undergone treatment for 24 h were tested for the protein expression of IRE1α and AFT4, with β-actin and total TSC2 as controls **D**.. PCR products of spliced and unspliced XBP1 following 6 h drug treatment were resolved on agarose gels (unspliced = 480 bp upper band, spliced = 454 bp lower band) **E**. and quantified in **F**.. * *p*<0.05, **p<0.01, ***p<0.001.

As both nelfinavir and salinomycin as single agents have been reported to induce ER stress in other cell lines [21, 25], we determined their impact alone and in combination in both *Tsc2*^−/−^ and *Tsc2*^+/+^ MEFs. To examine the relative levels of ER stress burden, a series of downstream ER stress markers were assessed after 6 h of drug treatment. mRNA levels of CCAAT-enhancer-binding protein homologous protein (CHOP), HSP70, and ER degradation enhancer mannosidase alpha-like 1 (EDEM1) were measured (Figure 1A-1C). Significantly higher basal gene expression of these ER stress markers was observed in the *Tsc2*^−/−^ Biostatus Ltd, Shepshed to wild-type cells, as previously reported [16, 31]. While nelfinavir enhanced mRNA expression in both cells, mRNA expression was elevated to a much higher level in the *Tsc2*^−/−^ MEFs. As a single agent, salinomycin induced an increase in *CHOP* expression in both cell lines, but had little impact on *HSP70* or *EDEM1* mRNA levels. Protein levels of IRE1α and ATF4 were also elevated upon single or combined treatment with nelfinavir and salinomycin (Figure 1D). To further assess ER stress, we examined XBP1 splicing (Figure 1E-1F). As part of the UPR during ER stress, IRE1α catalyses the excision of an unconventional intron within XBP1 mRNA that can be measured *via* PCR. In the *Tsc2*^−/−^ MEFs, the lower spliced XBP1 PCR product (loss of 26 nucleotides) is the predominant XBP1 mRNA form after nelfinavir treatment, while only 40 % (± 4 %) of XBP1 mRNA was spliced in the wild-type controls. Combined nelfinavir/salinomycin treatment caused a further 16 % increase in XBP1 splicing in wild-type cells, though could not further enhance the already high level of XBP1 splicing seen in the nelfinavir treated *Tsc2*^−/−^ MEFs. This data demonstrates that nelvinavir and/or salinomycin can induce a high level of ER stress in cells lacking *Tsc2*.

### Salinomycin synergises with nelfinavir to selectively kill Tsc2−/− MEFs

**Figure 2:**
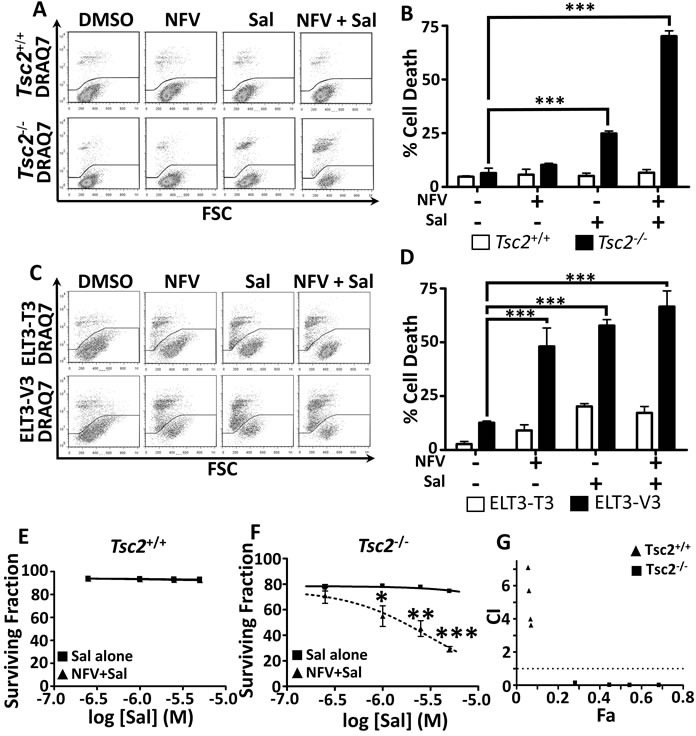
Dual nelfinavir/salinomycin treatment kills *TSC2*^−/−^ MEFs synergistically *Tsc2*^+/+^ and *Tsc2*^−/−^ MEFs were treated with either DMSO vehicle, 20 μM nelfinavir (NFV) or 5 μM salinomycin (Sal) as single agents or in combination for 24 h and cell death was assessed by flow cytometry using DRAQ7 **A**., quantified in **B**. ELT3-V3 and *TSC2*-re-expressing ELT3-T3 cells were similarly treated for 48 h and analysed for cell death **C**., **D**. Synergy was assessed by examining cell death across a range of salinomycin concentrations, with or without 20 μM nelfinavir **E**., **F**. and calculated using CompuSyn software **G**.. Graphs show an average of three independent replicates, mean +/− S.E.M. * *p* < 0.05, ** *p* < 0.01, *** *p* < 0.001.

Extended periods of ER stress can lead to cellular death if not efficiently restored [12]. To quantify cell death after drug treatments, we carried out flow cytometry with DRAQ7 (Figure 2A, graphed in Figure 2B). DRAQ7 is a membrane impermeable far-red fluorescent dye which, when membrane integrity is compromised, enters the cell and binds readily to nuclear DNA to report cell death [32, 33]. As a single agent, salinomycin induces a moderate percentage of cell death in the *Tsc2*^−/−^ MEFs (25 % ± 2 %), while no apparent cytotoxicity was observed in the wild-type controls. When nelfinavir was combined with salinomycin, we observed a dramatic induction of cell death in the *Tsc2*^−/−^ MEFs. Of interest, combined treatment with nelfinavir and salinomycin did not induce cell death in the *Tsc2*^+/+^ MEFs, which was equivalent to the DMSO treated controls (at about 5% in both cases). To further confirm that combined nelfinavir and salinomycin treatment induced cytotoxicity as a consequence of *Tsc2* loss, we utilised ELT3 cells. ELT3 cells are derived from an Eker rat uterine leiomyoma that are *Tsc2*-null (referred to as ‘ELT3-V3’). We compared these *Tsc2*-null cells to a TSC2 re-expressing rescued control cell line (referred to as ‘ELT3-T3’ [27]). As observed in the MEFs, ELT3 cells lacking *Tsc2* displayed a higher level of cell death, while the TSC2 re-expressing ELT3 cells better tolerated nelfinavir and salinomycin as either single treatments or when combined (Figure 2C-2D).

Having demonstrated that combined nelfinavir and salinomycin treatment is effective at killing cells lacking *Tsc2*, we next wanted to determine the property of drug synergy between nelfinavir and salinomycin. To do this, we treated both *Tsc2*^−/−^ and *Tsc2*^+/+^ MEFs with a broad range of drug concentrations of nelfinavir and salinomycin as single agents and in combination. Cell death was similarly assessed using flow cytometry and DRAQ7 labelling. The *Tsc2*^−/−^ MEFs were observed to be acutely sensitive to combined treatment with nelfinavir and salinomycin and at much lower drug concentrations (Figure 2F), while the *Tsc2*^+/+^ MEFs tolerated even the highest of drug concentrations (Figure 2E). CompuSyn software was used to calculate combination index (CI) values based on mean cell death and is presented as a Chou-Talalay plot (Figure 2G). CI values below 1 indicate synergistic drug action between nelfinavir and salinomycin. Importantly, we found that nelfinavir and salinomycin acted in a synergistic manner to kill *Tsc2*^−/−^ MEFs, but no synergy was observed in the *Tsc2*^+/+^ MEFs.

### Nelfinavir and salinomycin dual treatment kills cells in an mTORC1-dependent manner

**Figure 3:**
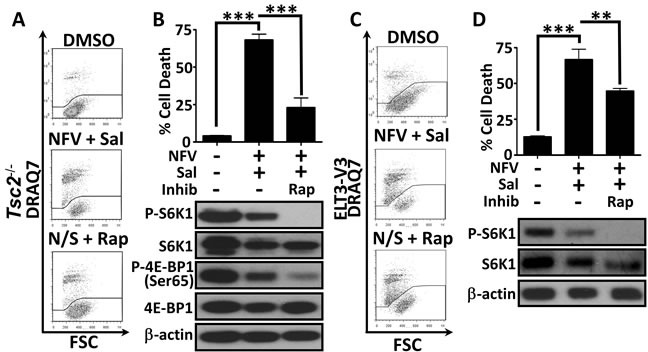
Dual treatment kills cells in a mTORC1-dependent manner *Tsc2*^+/+^ and *Tsc2*^−/−^ MEFs were treated with DMSO or a 20 μM nelfinavir (NFV) plus 5 μM salinomycin (Sal) combination for 24 h, with or without the mTOR inhibitor, rapamycin (Rap). Cell death was assessed by flow cytometry and mTORC1 inhibition was determined by western blotting for phospho-S6K1 (T389) and phospho-4E-BP1 (Ser65) **A**. and **B**. Similar analysis of ELT3 cells was performed following 48 h drug treatment, with phospho-S6K1 used as a readout of mTORC1 activity **C**. and **D**. Graphs show an average of three independent replicates, mean +/− S.E.M. **p<0.01, ***p<0.001.

To determine whether mTORC1 activation following *Tsc2* loss was responsible for the selective sensitivity of the cells to nelfinavir/salinomycin dual treatment (rather than *via* another TSC2 function), we used rapamycin (an allosteric mTORC1 inhibitor) to block mTORC1 signalling. Treatment with rapamycin substantially rescued cell death in the *Tsc2*^−/−^ MEFs (Figure 3A and graphed in Figure 3B). Inhibiting mTOR also partially rescued nelfinavir and salinomycin induced cell death in the ELT3-V3 cells (Figure 3C and graphed in Figure 3D). This data indicates that hyperactive mTORC1 contributes to nelfinavir and salinomycin-induced cytotoxicity.

### Nelfinavir and salinomycin induce cell death through an increased protein synthesis burden

**Figure 4:**
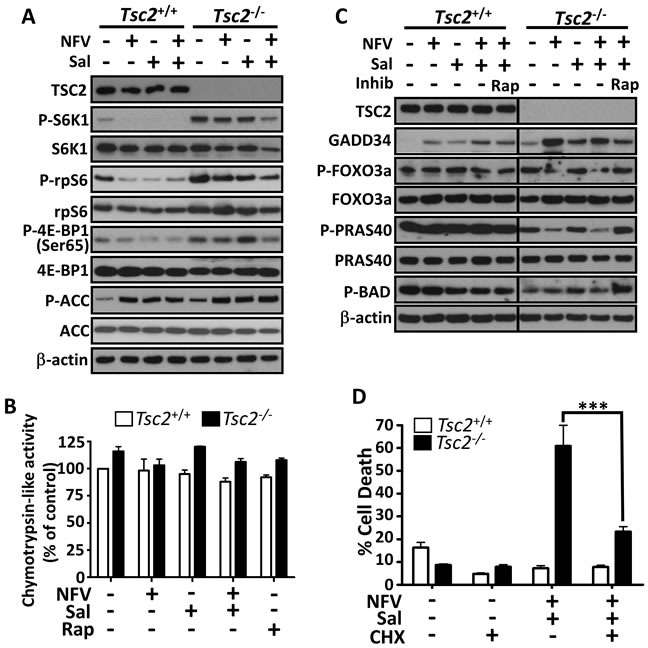
Mechanisms of nelfinavir/salinomycin-induced cell death Following a 24 h incubation with the indicated treatments, *Tsc2*^+/+^ and *Tsc2*^−/−^ MEFs were lysed and protein levels of phosphorylated S6K1, rpS6, 4E-BP1 and ACC were determined by western blot. Total TSC2, S6K1, rpS6, 4E-BP1, ACC and β-actin are shown as controls **A**.. The proteasome activity of drug treated samples was determined by monitoring the turnover of the fluorescent chymotrypsin-like substrate **B**.. Samples prepared as in A were further analysed for total GADD34, phospho-FOXO3a, phospho-PRAS40 and phospho-Bad. *Tsc2*^+/+^ and *Tsc2*^−/−^ samples were run on the same gel and are shown at the same exposure **C**.. *Tsc2*^+/+^ and *Tsc2*^−/−^ MEFs were pre-treated with cycloheximide (CHX) for 1 h, then treated with the indicated combinations for 24 h. Cell death was assessed by flow cytometry **D**.. *** *p* < 0.001.

Studies in multiple cell lines have implicated different mechanisms of salinomycin induced cell death [18–22]. To elucidate which processes may be involved in *Tsc2*-deficient cells, we firstly examined mTORC1 and AMPK signalling. We found that as single agents, the weak mTORC1 signalling in *Tsc2* wild-type cells could be inhibited, but neither drug alone impacted substantially on S6K1, rpS6 or 4E-BP1 phosphorylation in *Tsc2*-deficient cells. The combination could reduce both phospho-4E-BP1 and phospho-S6K1 levels in *Tsc2*-deficient cells after 24 h, together with the downstream substrate, phospho-rpS6 (Figure 4A and also seen in Figure 3B). Interestingly, the dual treatment also impacted the total levels of S6K1, but only in the *Tsc2*-deficient cells. AMPK activity, as measured *via* phospho-ACC, was elevated following single or combination treatments (Figure 4A), indicating enhanced energy stress following treatment. Nelfinavir has previously been shown to impair the proteasome [34], whereas salinomycin's action appears to be independent of the proteasome [18]. To assess how the drug combination impacted the proteasome in our cells, we measured the proteasomal chymotrypsin-like activity (Figure 4B). We generally observed a higher level of proteasome activity in the *Tsc2*^−/−^ MEFs, but there were no significant changes to activity upon drug treatment, indicating that nelfinavir/salinomycin-induced cell death is independent of the proteasome. To determine if cell death involved the classical caspase cascade, we analysed cleavage of PARP and caspase 3. Salinomycin caused mild PARP and caspase-3 cleavage in the *Tsc2*^−/−^ MEFs as a single agent that was not further enhanced upon nelfinavir treatment (Supplementary Figure 1). This suggests an alternative, non-classical cell death mechanism is largely responsible for nelfinavir/salinomycin-induced cytotoxicity.

Levels of GADD34, which is upregulated in response to ER stress, have been shown to correlate with cell death [35] and GADD34 overexpression reduces cell viability when combined with irradiation [36]. Therefore, we assessed GADD34 levels in *Tsc2* MEFs following drug treatment. We found that nelfinavir strongly enhanced GADD34 expression, in keeping with an induction of ER stress (Figure 4C). While salinomycin modestly enhanced GADD34 levels as a single agent, combination treatment showed a higher level of GADD34 expression than with salinomycin alone. While increased GADD34 did not completely correlate with cell death in *Tsc2* MEFs, we were interested to observe that rapamycin pre-treatment of the cells prevented the upregulation of GADD34 seen in dual nelfinavir/salinomycin treated cells. This could indicate that the reduction in cell death following rapamycin treatment (Figure 3) is due to a reduced burden of ER stress in these cells. In neurons, it was found that GADD34 could reduce Akt phosphorylation [37]. As Akt activation and the downstream phosphorylation of proteins such as Foxo3a and BAD is viewed as pro-survival, we assessed FOXO3a phosphorylation at the S256 Akt site. We found that phospho-FOXO3a levels showed the inverse pattern to GADD34 levels, being reduced in *Tsc2*^−/−^ MEFs following combination nelfinavir/salinomycin treatment and enhanced when cells were pre-treated to inhibit mTORC1 prior to nelfinavir/salinomycin treatment (Figure 4C). Similarly, the Akt-induced phospho-PRAS40 site (Thr246) and phospho-BAD site (Ser136) were increased with rapamycin pre-treatment compared to nelfinavir/salinomycin treatment alone. This suggests that high ER stress induced through nelfinavir/salinomycin treatment of mTORC1 hyperactive cells correlates with a decrease in pro-survival Akt signalling, which could be one of the mechanisms by which the treatments induce cell death.

The ER stress responsive proteins, ATF4 and CHOP, have been shown to cause increased protein synthesis, leading to a reduction in cell survival [38]. As we observed nelfinavir and salinomycin enhanced *CHOP* mRNA expression (Figure 1A) and ATF4 protein levels (Figure 1D), we investigated whether the enhanced burden of protein synthesis present in *Tsc2*-deficient cells combined with the treatment-induced elevation of CHOP and ATF4 could play a role in causing cell death. Pharmacological inhibition of protein synthesis using cycloheximide substantially rescued (> 60 %) nelfinavir/salinomycin induced *Tsc2*-deficient cell death (Figure 4D), which was comparable to rapamycin (Figure 3).

### Nelfinavir and salinomycin show cytotoxity towards Tsc2-null tumour spheroid models while rapamycin acts as a cytostatic agent

**Figure 5:**
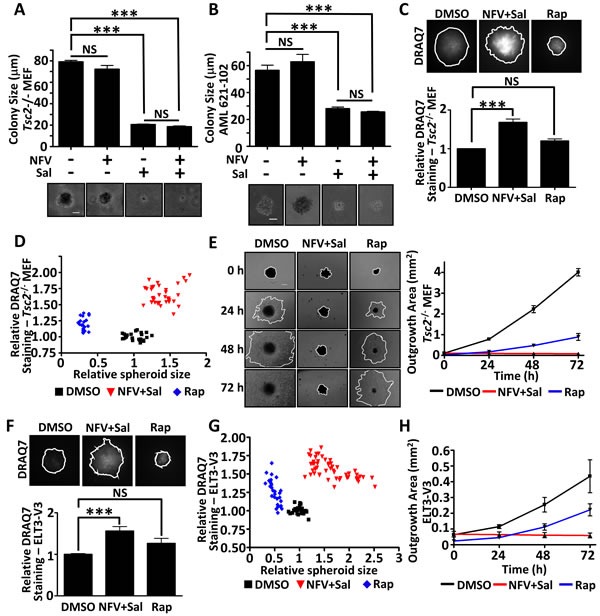
Dual nelfinavir/salinomycin treatment is effective in 3D models *Tsc2*^−/−^ MEFs **A**. or *TSC*-null AML cells **B**. were grown in soft agar and treated with DMSO, 10 μM nelfinavir (NFV), 2 μM salinomycin (Sal) or a NFV/Sal combination for 11 days. Images of the colonies were taken and the diameters measured using Image J. The scale bar represents 50 μm. *Tsc2*^−/−^ MEFs were allowed to self-aggregate into spheroids under non-adherent conditions and were treated with DMSO, 10 μM nelfinavir (NFV) plus 2 μM salinomycin (Sal) in combination or 25 nM rapamycin (Rap), as indicated, for 96 h. DRAQ7 was added for the final 36 h to monitor cell death. DRAQ7 images were taken and quantified **C**.. Spheroid diameter was determined from phase contrast images taken after 96 h drug treatment and plotted against DRAQ7 staining intensity **D**.. Spheroids were then replated onto standard tissue culture plates and allowed to grow under drug free conditions. Images were taken every 24 h and the area of outgrowth calculated using Image J **E**.. The scale bar represents 200 μm. Spheroid experiments were repeated in ELT3-V3 cells (F-H). ***p<0.001, NS = not significant.

To determine whether the drug combination was successful in a 3D setting, we next tested nelfinavir and salinomycin within *Tsc2*-deficient tumour spheroid model systems. We observed that salinomycin as a single agent could markedly block tumour spheroid formation of *Tsc2*^−/−^ MEFs in soft agar (Figure 5A) as well as TSC-patient derived AML cells that also lack *Tsc2* (Figure 5B). Combined treatment of salinomycin with nelfinavir was equally as potent at reducing tumour formation. Due to the longer time period of the experiment compared to the monolayer cultures, we found that lower concentrations of both drugs were effective at causing cell death in our 3D systems. We next wanted to assess whether established spheroids were also sensitive to nelfinavir and salinomycin. To do this, *Tsc2*^−/−^ MEF tumour spheroids were allowed to form over 72 h and then treated for 4 days with either DMSO, nelfinavir/salinomycin, or rapamycin. Spheroids were stained with DRAQ7 to measure the relative levels of cell death (Figure 5C). We observed distinct tumour spheroid populations after both nelfinavir/salinomycin or rapamycin treatments, with marked differences in DRAQ7 staining intensity as well as relative spheroid area (Figure 5D). While rapamycin caused a marked reduction in spheroid size, there was little apparent cell death, as observed by a slight increase in DRAQ7 staining that was not significantly different to untreated cells (DMSO). In contrast to rapamycin, dual nelfinavir and salinomycin treatment caused a slight increase in spheroid size due to the development of a more uneven spheroid surface. Furthermore, there was a significant level of cell death after nelfinavir and salinomycin treatment, as observed by a high level of DRAQ7 staining within the tumour core (Figure 5C). After cessation of drug treatment and re-plating into adherent plates, we measured outgrowth of cells from these spheroids (Figure 5E). This provided another readout of tumour recovery and long-term pharmacodynamics consequence of the original drug treatment. Of note, we observed cell outgrowth from spheroids that were previously treated with rapamycin, revealing a significant degree of cell recovery. This property, combined with low levels of cell death, indicates that rapamycin is acting as a cytostatic agent. As no cell outgrowth was observed over the 72 h period from the spheroids previously treated with nelfinavir and salinomycin, this drug combination has cytotoxic properties throughout the entire spheroid. This experiment was repeated using ELT3-V3 cells (Figure 5F-5H), revealing an identical pattern and supporting the view that nelfinavir/salinomycin is a cytotoxic combination to *TSC2*-deficient spheroids.

### Sporadic cancers with hyperactive mTORC1 are also killed by dual nelfinavir/salinomycin treatment

**Figure 6:**
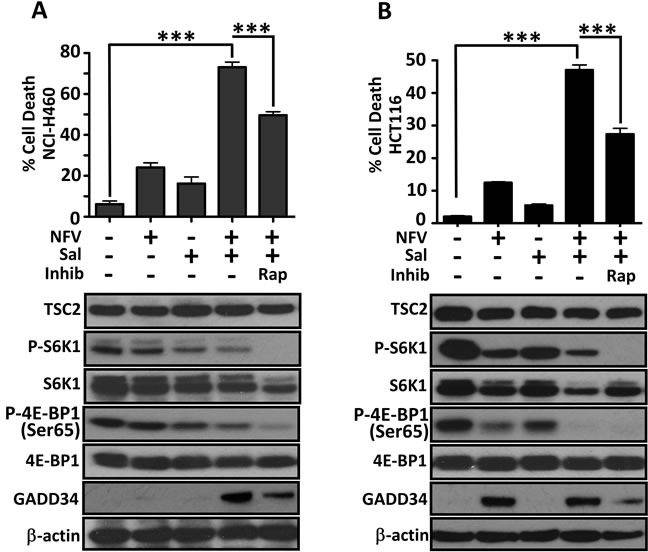
Sporadic cancer cell lines with hyperactive mTORC1 are also sensitive to nelfinavir and salinomycin Cell death of NCI-H460 cells was determined by flow cytometry following 48 h incubation with 20 μM nelfinavir (NFV) and/or 5 μM salinomycin (Sal), with or without 1 h rapamycin (Rap) pre-treatment **A**.. Cell lysates collected after 24 h treatment were analysed for mTORC1 activation (phosphorylated S6K1 and 4E-BP1) and total GADD34 levels **A**.. Experiments were repeated in HCT116 cells **B**.. *** *p* < 0.001.

As our data indicates that blockade of mTORC1 signalling allows cells to evade nelfinavir/salinomycin-induced death, it follows that other cell types with high levels of mTORC1 activity should also be sensitive to the drug combination. To test this concept further, we utilised two cell lines with basally high mTORC1 signalling (Figure 6): the NCI-H460 large cell lung cancer cell line which has *PI3KCA*, *CDKN2A*, *STK11* and *KRAS* mutations [39] and the HCT116 colorectal cancer cell line, which contains an activating *RAS* mutation [40] and *PIK3CA* mutation [41]. In both cell lines, dual nelfinavir/salinomycin treatment significantly enhanced cell death. The addition of rapamycin to combined treatment rescued 33-57 % of cell death (Figure 6A-6B). We also observed a strong induction of GADD34 expression in both cell lines with dual nelfinavir/salinomycin treatment, which was substantially reduced with rapamycin treatment. This implies that an increased ER stress burden may contribute to cell death in these mTORC1 hyperactive sporadic cancers.

## DISCUSSION

Our work shows that targeting the ER stress vulnerability of mTORC1 hyperactive cells has promise for the eradication of such cells in Tuberous Sclerosis Complex and cancer. We found that nelfinavir and salinomycin act synergistically to selectively kill *Tsc2*-null MEFs, while normal cells with intact *Tsc2* tolerate treatment (Figure 2). Cytotoxicity is not through the classical apoptotic caspase cascade as minimal PARP and caspase 3 cleavage were observed following dual drug treatment. This fits with a previous report where a non-classical mechanism of cell death was observed following salinomycin treatment in other cell types [18]. Additionally, as *Tsc2* MEFs are *TP53*-null, our data indicates that nelfinavir/salinomycin induced cell death is independent of p53, fitting with a previous study where salinomycin showed cytotoxic properties in *TP53*-null Jurkat cells [18]. Salinomycin's mode of action is also not through proteasomal inhibition, as it did not impact proteasomal turnover (Figure 4B), supporting the findings of others [18].

Regulation of protein synthesis and cell death have been linked previously [38, 42], with ATF4 and CHOP reported to have important roles. A block in protein synthesis through knockdown of ribosomal genes or pharmacological inhibition has been shown to increase cell viability in ATF4/CHOP over-expressing cells [38]. When we inhibited protein synthesis by downregulating mTORC1 signalling with rapamycin (Figure 3) or by using cycloheximide (Figure 4D), we were able to significantly rescue nelfinavir/salinomycin induced cell death. Other studies have shown that increased protein synthesis leads to ATP depletion [38] and in accordance with this, we observe an elevation of AMPK signalling following nelfinavir/salinomycin dual treatment (Figure 4A). We also observed an increase in phosphorylation of the pro-survival Akt substrates FOXO3a, PRAS40 and BAD [43], together with a reduction in ER stress burden, as measured by GADD34 expression, when mTORC1 was inhibited in *TSC2*-null MEFs prior to dual nelfinavir/salinomycin treatment (Figure 4C). This suggests that high protein synthesis resulting in low cellular energy levels and decreased survival signalling could be one pathway responsible for sensitising *TSC2*-null cells to nelfinavir/salinomycin induced death.

There is much interest in the potential use of salinomycin in humans following its identification as an agent which could target cancer stem cells [17]. Although a review article highlighted two promising case reports of its use in cancer patients who had exhausted other therapeutic options [44], salinomycin is unlikely to rapidly translate to clinical use as there is a lack of mammalian safety data and previous reports indicate severe toxicity following accidental human exposure [45]. While some new salinomycin esters and diastereoisomers have been synthesised which also exhibit anti-cancer properties [46, 47], they still show evidence of neurotoxicity in mice. Currently, the specific protein target of salinomycin is unknown. Should it be uncovered, it may be possible to target the signalling pathway with an alternative drug, which in combination with nelfinavir, could prove to be a clinically viable combination with specificity for killing mTORC1 hyperactive cells.

In this study, 3D colony formation and spheroid assays permitted us to determine whether the nelfinavir/salinomycin induced cell death we observed in monolayer cell culture could be replicated in a more physiological 3D model, an important step in pre-clinical drug testing. We found salinomycin either alone, or in combination with nelfinavir, effectively blocked colony formation in soft agar (Figure 5A-5B). Rapalogues, the current therapy for TSC, result in a cytostatic rather than a cytotoxic clinical response. Our 3D spheroid system depicts this shortcoming of using rapamycin to treat TSC patient tumours, as growth recovery was apparent upon its removal, showing that rapamycin is a cytostatic agent (Figure 5). In contrast to rapamycin, dual nelfinavir/salinomycin treatment caused complete tumour death (Figure 5). This data clearly shows that the nelfinavir and salinomycin drug combination is highly cytotoxic to mTORC1 hyperactive cells.

This work also highlights the essential steps of using 3D culture models in dissecting the pharmacodynamic consequences on TSC tumours. The nelfinavir/salinomycin combination did not shrink the spheroid size, unlike rapamycin, and so in other tumour models may have been discarded as an ineffective therapy. However, by embedding the non-toxic viability label, DRAQ7, directly into the treatment timecourse, combined with the additional validation of the outgrowth assay, it was clear that the cells forming these spheroids were no longer viable. In contrast, the rapamycin treated spheroids immediately began to regrow following drug removal. This spheroid assay shows the importance of discriminating between cytostatic treatments and those which are likely to bring about a long-lasting cytotoxic response, making it an innovative and useful screening tool for future TSC therapies.

## MATERIALS AND METHODS

### Tissue culture and drug treatments

*Tsc2*^+/+^ *p53*^−/−^ and *Tsc2*^−/−^ *p53*^−/−^ mouse embryonic fibroblasts (MEFs) were a kind gift from David Kwiatkowski (Harvard University, Boston, USA) in 2004 and have been previously characterised [26]. Eker rat leiomyoma-derived *Tsc2*-deficient ELT3-V3 cells and matching control *TSC2*-expressing ELT3-T3 cells generated in Astrinidis et al, 2002 [27], were kindly provided in 2006 by Cheryl Walker (M.D. Anderson Cancer Center, Houston, USA). Human lung carcinoma (NCI-H460) cells were purchased from ATCC in 2012 while HCT116 cells were provided in 2015 by Nick Leslie (Heriot Watt University, Edinburgh). All cell lines were mycoplasma free and regularly tested for mycoplasma infection using the Venor GeM Classic PCR kit from CamBio. Cells were cultured in Dulbecco's Modified Eagle's Medium (DMEM), supplemented with 10 % (v/v) foetal bovine serum (FBS), 100 U/ml penicillin and 100 μ/ml streptomycin (Life Technologies Ltd., Paisley, UK) in a humidified incubator at 37°C, 5 % (v/v) CO_2_. Angiomyolipoma (AML) cells 621-102 were a kind gift from Elizabeth Henske (Brigham and Women's Hospital, Boston, U.S.A.) in 2013 and were cultured as above, but in media containing 15% FBS. Cell lines were cultured for no more than 3 months following their initial thawing. Nelfinavir mesylate hydrate, salinomycin (ready-made solution) and rapamycin were purchased from Sigma-Aldrich Company Ltd. (Dorset, UK). Nelfinavir and rapamycin were dissolved in dimethyl sulfoxide (DMSO) and further diluted in culture medium to the required concentrations before use. Nelfinavir and salinomycin concentrations were chosen based on solubility and dose response curves carried out in *Tsc2* MEFs.

### Western blotting and antibodies

Cells were lysed in cell lysis buffer (20 mM Tris, pH 7.5, 135 mM NaCl, 5% [v/v] glycerol, 50 mM NaF, 0.1% [v/v] Triton X-100, plus protease inhibitors), centrifuged and protein quantified using Bradford reagent (Sigma-Aldrich). Samples were made up in NuPAGE LDS sample buffer (Life Technologies). Western blotting was performed as previously described [28]. Blots shown are representative of 3 independent experiments. Anti-β-actin (#4967), phospho-TSC2 S1387 (#5584), total TSC2 (#3990), IRE1α (3294S), phospho-S6K1 T389 (#9205), total S6K1 (#9202), phospho-rpS6 S235/236 (#2211), total rpS6 (#2217), phospho-4E-BP1 S65 (#9451), total 4E-BP1 (#9644), phospho-ACC S79 (#3661), total ACC (#3676), PARP (#9542), caspase 3 (#9662), ATF4 (#11815), phospho-FOXO3a (#9466S), total FOXO3a (#9467), phospho-PRAS40 T246 (#2997), total PRAS40 (#2691) and phospho-Bad S136 (#4366) antibodies were from Cell Signaling Technology (Danvers, MA, USA). GADD34 antibody (10449-1-AP) was from Proteintech (Manchester, UK).

### RNA extraction, Q-PCR and XBP1 splicing

This was performed as described previously [16] with the additional use of *HSP70* and *EDEM1* real time Quantitect PCR primers which were obtained from Qiagen (West Sussex, United Kingdom).

### Flow cytometry

To measure cell death prevalence in the population, drug treated cells were trypsinised from 24 well plates and incubated with 3 μM DRAQ7^TM^ (Biostatus Ltd, Shepshed, UK) for 10 min at 37°C. Flow cytometry was performed using a FACS Calibur flow cytometer (Becton Dickinson, Cowley, UK) using excitation at 488 nm and detection of fluorescence in log mode at wavelengths greater than 695 nm (far red). Cell Quest Pro software (Beckton Dickinson Immunocytometry Systems) was used for signal acquisition. Autofluorescence of unstained cells provided the background readout across all samples. Correlated signals were collected for a minimum of 10,000 events. Events were gated and analysed using FlowJo software (Oregon, USA).

### Synergy analysis using compuSyn

Cell viability obtained through flow cytometry analysis was inputted into CompuSyn software (ComboSyn, Inc, [29]) to obtain Combination Index (CI) values using a non-constant ratio approach. Flow cytometry values used were the mean of three independent experiments.

### Proteasome assay

Proteasomes were extracted from live cells 2 h post-treatment according to a previously described protocol [30]. Chymotrypsin-like proteasome activity was assessed using the fluorogenic substrate Succ-LLVY-AMC (Sigma). Assays were performed as described (Crawford et al, 2006), with the rate of substrate turnover determined by monitoring the fluorescence of released aminomethylcoumarin using a multiwell plate reader (FLUOstar Optima, BMG Labtech) at an excitation wavelength of 395 nm and emission wavelength of 460 nm over a period of 35 min.

### Soft agar assays

Two-layered soft agar assays were performed in 6 well plates using a base layer of 0.6 % agar in standard culture media. *Tsc2*^−/−^ MEFs or AML 621-102 cells (1.5 × 10^5^ cells per well) were suspended in a top layer of 0.3 % (w/v) agar. Complete media containing the indicated treatments was added the following day. Cultures were grown for 11 days, with media (and drug treatments) replaced every 2-3 days. Images were taken using an EVOS XL Core camera (Life Technologies).

### 3D spheroids and outgrowth

*Tsc2*^−/−^ MEFs or ELT3-V3 cells were plated at 1000 cells/well into a 96-well plate, precoated with 1.5 % (w/v) agarose. Spheroids were allowed to form for 72 h before treatment with DMSO, 10 μM nelfinavir and 2 μM salinomycin or 25 nM rapamycin. Fresh media and drugs, including DRAQ7 (which is non-toxic), were added by removal and replacement of 50 % of the media volume after 60 h, and incubated for a further 36 h (total 96 h treatment incubation). Dual channel images were acquired using a Zeiss Axio Observer Z1 microscope (Carl Zeiss Microimaging, Gottingen, Germany) with a black box chamber (Solent Scientific Ltd, Segensworth, U.K.) at 0 and 96 h timepoints. Spheroid size (transmission mode) and DRAQ7 labelling (fluorescence excitation 488nm/emission above 695nm) were assessed using MetaMorph acquisition software. Following imaging, spheroids were transferred to a standard, tissue culture coated 24 well plate with fresh culture media (no drug treatments) and imaged using an EVOS XL Core camera (Life Technologies) after 0, 24, 48 and 72 h. Total outgrowth area from the spheroid was measured using ImageJ (v1.50i) software (https://imagej.nih.gov/ij/).

### Statistical analysis

All reported flow cytometry and soft agar assay results are from three independent experiments. Proteasome assays were independently performed three times in duplicate. 3D spheroid data was from an average of 10 spheroids per condition and repeated across three independent experiments. All statistical analyses were conducted using SPSS20 (IBM). Data are graphed as the mean ± SEM. Multiple data sets were compared using one way ANOVA, followed by the LSD post-hoc test.

## SUPPLEMENTARY FIGURE



## Abbreviations

AMPK: 5′ AMP-activated protein kinase
ATF4: activating transcription factor 4
CHOP: C/EBP homologous protein
CI: combination index
DMSO: dimethyl sulfoxide
EDEM1: ER degradation enhancer mannosidase alpha-like 1
ER: endoplasmic reticulum
GADD34: growth arrest and DNA damage-inducible protein 34
FOXO3a: Forkhead box O3a
HIV: human immunodeficiency virus
HSP: heat shock protein
MEF: mouse embryonic fibroblast
mTORC1: mechanistic target of rapamycin complex 1
PRAS40: proline-rich Akt substrate of 40 kDa
rpS6: ribosomal protein S6
S6K1: S6 kinase 1
TSC: tuberous sclerosis complex
UPR: unfolded protein response
