# Supplementary material for: Targeting protein homeostasis with nelfinavir/salinomycin dual therapy effectively induces death of mTORC1 hyperactive cells
Source: Oncotarget. 2017 Mar 15;8(30):48711–24. doi: 10.18632/oncotarget.16232 (PMC5564719; doi:10.18632/oncotarget.16232)
Supplement: Supplementary file 1 [file oncotarget-08-48711-s001.pdf]

Targeting protein homeostasis with nelfinavir/salinomycin dual therapy effectively induces death of mTORC1 hyperactive cells

Supplementary Materials

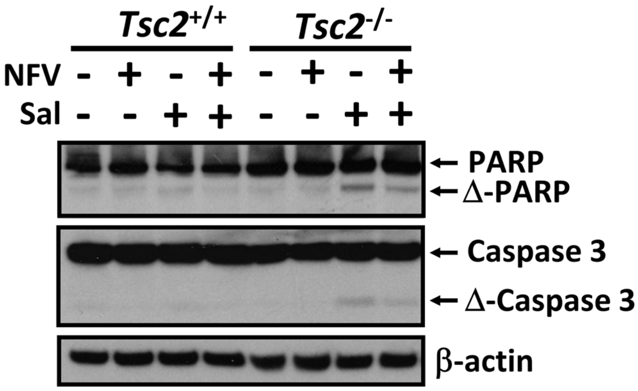

Supplementary Figure 1: The classical apoptotic cascade is minimally activated in response to nelfinavir and salinomycin. Samples from Figure 4A were probed for PARP and caspase 3 cleavage.
